# Pumping iron

**DOI:** 10.7554/eLife.03997

**Published:** 2014-08-15

**Authors:** Caroline C Philpott

**Affiliations:** 1 **Caroline C Philpott** is in the National Institutes of Diabetes and Digestive and Kidney Diseases, National Institutes of Health, Bethesda, United States

**Keywords:** Slc39a13, iron transporter, secretory compartments, Ehlers-Danlos syndrome, Drosophila, metal transport, *D. melanogaster*

## Abstract

The primary role of the ZIP13 metal transporter in flies is to move iron ions out of cells, rather than moving zinc ions into cells, as is the case in human cells.

**Related research article** Xiao G, Wan Z, Fan Q, Tang X, Zhou B. 2014. The metal transporter ZIP13 supplies iron into the secretory pathway in *Drosophila melanogaster*. *eLife* **3**:e03191. doi: 10.7554/eLife.03191

**Image** Staining (blue) shows the location of iron in the anterior midgut of a fruit fly larva

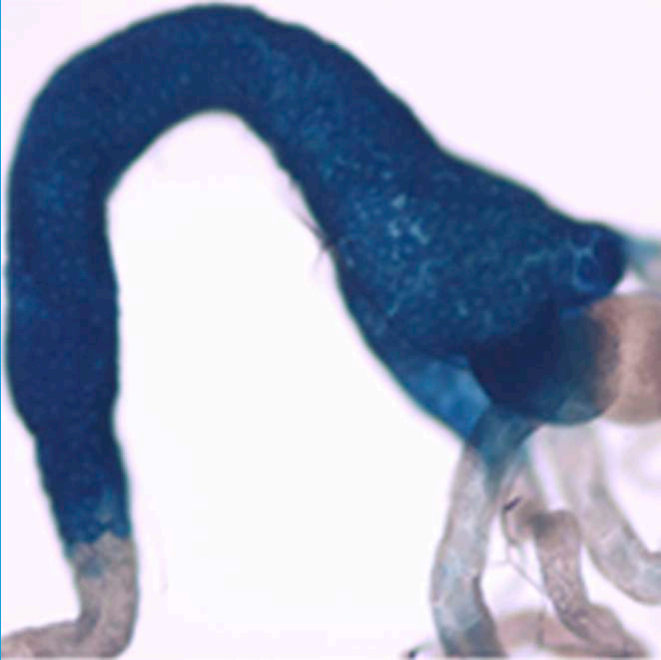

Thousands of proteins must bind to metal ions in order to function properly. Maintaining healthy amounts of metals in cells should be simple: cells have access to a large pool of metal ions and all they need to do, in principle, is to select the metal ions they need from this pool. The reality, however, is different: the pool of available metals is really an alphabet soup of essential nutrients, trace elements and deadly toxins, and the availability of the metals in this pool in no way matches the nutritional requirements of the organism. Cells must, therefore, take up and distribute the correct amounts of each metal they need, notably iron and zinc, but also many others.

Because they have an electric charge, metal ions cannot enter cells by simple diffusion, and instead require dedicated transport systems to help them cross cell membranes. Metal ion transporters are a diverse group of proteins embedded in the plasma membrane, or in the membranes of the organelles inside the cell, that can transport specific metal ions into and out of the cytosol.

Metal transporters can be grouped into families based on their amino acid sequence. All the members of a given family tend to transport similar metal ions, and usually pump ions in only one direction—either into or out of the cell. Now, in *eLife*, Bing Zhou and co-workers from Tsinghua University—including Guiran Xiao and Zhihui Wan as joint first authors—have found that a transporter called ZIP13 transports iron out of cells in fruit flies (Xiao et al., 2014).

High levels of nutrient metals can be toxic, so the activity of metal ion transporters is highly regulated. Sensors detect the levels of metal inside the cell, and in turn control the production and destruction of the transporters. However, this approach ultimately runs into an inherent limitation; the transporters themselves have a limited capacity to discriminate between different metal ions.

In the case of the ZIP family of metal ion transporters, the name—ZIP is short for Zrt-, Irt-like protein—is the first indication of this limited selectivity. Zrt proteins were first identified as zinc importers in a species of yeast (Zhao and Eide, 1996). Soon afterwards, a similar gene, Irt1, descended from an ancestor of Zrt, was identified in the plant species *Arabidopsis thaliana* as an iron importer (Eide et al., 1996). Subsequently, ZIP-family transporters that can transport iron and zinc have been identified in essentially all eukaryotic genomes, with more complex eukaryotes expressing many different copies of ZIP-family genes (Lye et al., 2012; Jeong and Eide, 2013).

Further study has demonstrated that, in addition to zinc and iron, many ZIP-family transporters can also transport other metal ions, such as manganese, copper, nickel and cadmium (Dempski, 2012). The biological role of the ZIP gene may also be determined by the conditions under which it is expressed: for example, zinc-transporting ZIPs are expressed under zinc-deficient conditions.

In humans and mice, a ZIP gene called Zip13—which is thought to specifically transport zinc—has been studied in detail because mutations in humans are associated with a form of Ehlers-Danlos syndrome, a connective tissue disorder (Giunta et al., 2008; Bin et al., 2011; Jeong et al., 2012). Now, Xiao, Wan and co-workers have examined dZip13—the fly gene most closely related to the human Zip13 gene—and found that it primarily transports iron rather than zinc.

Comparing flies depleted of dZip13 with wild type flies revealed that the depleted flies have a lower total amount of iron in their bodies, but normal amounts of zinc. The depleted flies are also less likely to emerge from the pupal stage than normal, and have shortened life spans. Both of these symptoms are improved by treating the flies with iron supplements. However, treating the flies with zinc supplements has no effect.

Given the numerous roles that ZIP family members play in iron transport, this is not very surprising. What is more remarkable is the evidence that dZip13 transports iron out of cells. This evidence came from vesicles called microsomes, which form from the membranes of the organelles that make up the secretory compartments, where proteins that are to be released from the cell are made.

Microsomes from flies lacking dZip13 exhibited reduced iron uptake rates. As an accumulation of iron within microsomes represents a flux of iron out of the cytosol, this suggests that dZip13 transports iron out of the cell. Similarly, microsomes that overexpressed dZip13 showed increased rates of iron uptake, suggesting an increased rate of iron transport out of the cell. Additionally, dZip13 was detected on intracellular vesicles when the transport protein was introduced to a human cell model. Combining this evidence led Xiao, Wan and colleagues to postulate that dZip13 primarily moves iron from the cytosol to the secretory compartment (Figure 1). There, the iron can be used to load ferritin, an iron-transporting protein in the fly circulatory system. This could be the solution to the long-standing mystery about how iron is moved from the cytosol to the secretory pathway in flies.

**Figure 1.**
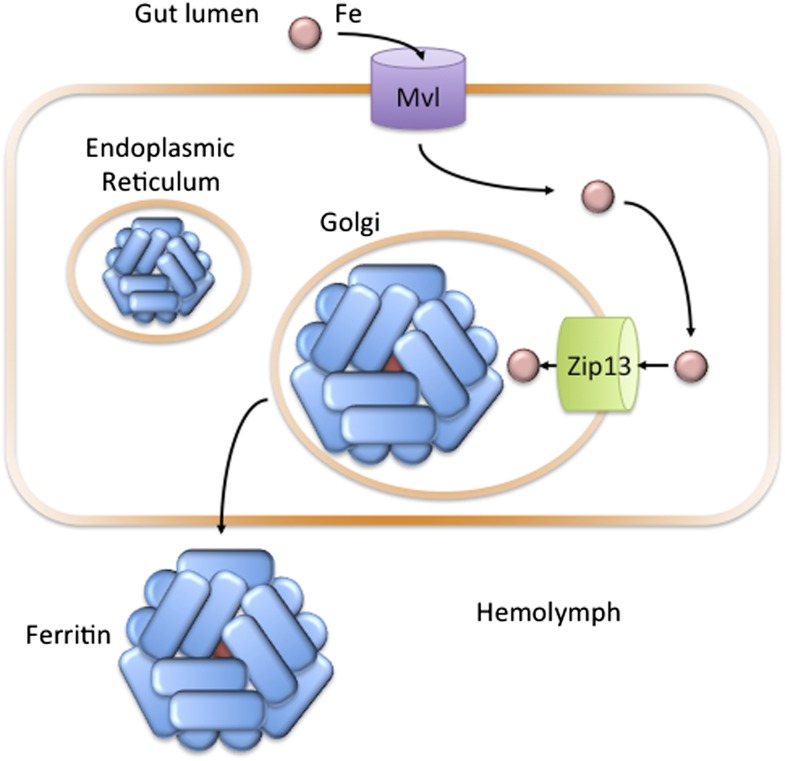
In the fruit fly *Drosophila melanogaster*, Zip13 transports iron into the secretory compartment. Iron (pink circle) present in the diet of the fly is transported from the gut lumen into cells that line the gut (orange rectangle). Iron uptake is likely controlled by the Fe(II) transporter Mvl, which is similar to the human iron transporter DMT1. Iron in the cytosol may then be transported across the membranes of the endoplasmic reticulum or Golgi apparatus to the lumen of the secretory pathway: Xiao, Wan et al. have found that a protein called Zip13 is responsible for transporting the iron ions across these membranes. Once in the secretory pathway, the ions can be incorporated into the protein ferritin, which is then secreted into the hemolymph to carry iron to other cells in the fly (Mandilaras et al., 2013).

Is there precedent for ZIP transporters transporting ions in both directions? In yeast, genetic evidence suggests that one particular ZIP transporter may move zinc both into and out of vesicles inside the cell, depending on the amount of zinc present in the cytosol (Kumanovics et al., 2006). Several other families of transporter have also been shown to mediate both the uptake and release of metal ions from cells (Pao et al., 1998; Rees et al., 2009; Vert et al., 2009).

What do these results mean for the human disease related to Zip13 mutation? There is strong evidence that Zip13 primarily transports zinc—not iron—when expressed in human cells, and the data presented here do not refute those studies. Fly dZip13 is 45% identical to the human version. Expression of human ZIP13 in flies partially, but not completely, compensates for the loss of fly ZIP13. This suggests that human Zip13 may be able to transport iron to a limited extent.

Despite the genetic similarities and the common ancestor the fly and human transporters share, they may differ in their ability to transport particular metal ions. Perhaps the capacity to operate as both an importer and an exporter, to localize to different subcellular membranes, and to recognize multiple divalent metal cations, explains the evolutionary push to maintain so many copies of the ZIP proteins in eukaryotic genomes.
